# Unmasking Latent Autoimmune Diabetes: A Case That Challenges Type 2 Diabetes

**DOI:** 10.7759/cureus.103361

**Published:** 2026-02-10

**Authors:** Nicholas W Tyndall, Franchesca Farris-Cosme, Dylan D Walker, Trevor I Colwell

**Affiliations:** 1 Internal Medicine, Brooke Army Medical Center, San Antonio, USA; 2 Internal Medicine, San Antonio Uniformed Services Health Education Consortium, San Antonio, USA

**Keywords:** diabetes type 2, elevated hba1c, euglycemia diabetic ketoacidosis, sodium glucose cotransporter-2 (sglt-2), type i diabetes mellitus

## Abstract

Latent autoimmune diabetes in adults (LADA) is an underrecognized form of autoimmune diabetes that is commonly misdiagnosed as type 2 diabetes mellitus (T2DM) because of its indolent progression and overlapping features with metabolic syndrome. We present a case of a 60-year-old woman with an initial diagnosis of T2DM who persistently had elevated hemoglobin A1c (HbA1c) despite optimal treatment and lifestyle modifications. After subsequent hospitalization and an episode of euglycemic diabetic ketoacidosis (DKA), further evaluation unmasked the diagnosis of LADA. This case emphasizes the need for a high index of suspicion for adults with poor glycemic control or unexplained DKA, as a timely diagnosis can significantly alter management and improve outcomes.

## Introduction

Latent autoimmune diabetes in adults (LADA) is a slowly progressive form of autoimmune diabetes that presents in adulthood and is often misclassified as T2DM [1]. Patients may initially respond to oral hypoglycemic agents but eventually require insulin therapy due to progressive beta-cell failure [2]. Misdiagnosis can lead to suboptimal glycemic control and an increased risk of complications, including diabetic ketoacidosis (DKA) [3]. We present the case of a 60-year-old woman initially diagnosed with T2DM who developed euglycemic DKA while receiving sodium glucose cotransporter-2 (SGLT-2) inhibitor therapy. Despite adherence to therapy, she had persistent hyperglycemia, prompting evaluation for LADA. This case emphasizes the risk of euglycemic DKA in adults with unrecognized autoimmune diabetes treated with SGLT-2 inhibitors and highlights the need for earlier consideration of autoantibody testing in patients presumed to have T2DM who demonstrate atypical features or poor glycemic response.

## Case presentation

**Figure 1:**
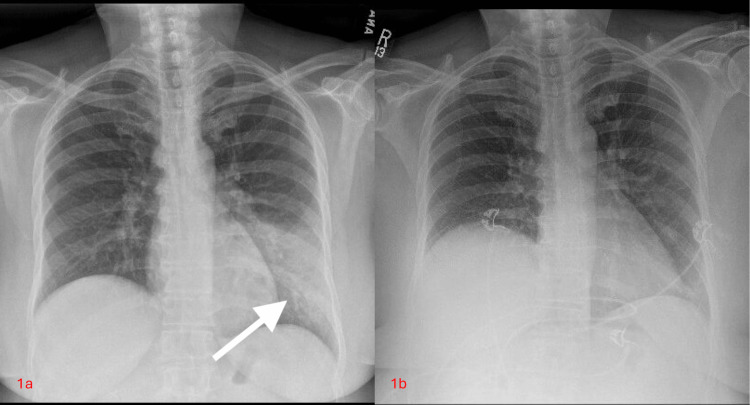
(a) Admission chest radiograph demonstrating left lower lobe consolidation. (b) Prior baseline chest radiograph for comparison.

A 60-year-old female with a past medical history of asthma, hyperlipidemia, type 2 diabetes mellitus (T2DM), metabolic syndrome, and a BMI of 32.4 was admitted to the intensive care unit for the management of euglycemic DKA. This admission occurred three weeks after hospitalization for acute hypoxemic respiratory failure secondary to influenza A. After recovery from the viral illness, she developed fever, fatigue, malaise, and a productive cough. A chest radiograph revealed left lower lobe consolidation consistent with pneumonia compared to previous imaging (Figures 1a-1b).

In the emergency department, the initial labs shown in Table 1 revealed a high anion gap metabolic acidosis with a gap of 25, a pH of 7.2, a blood glucose of 140, and moderate blood ketone levels, confirming the diagnosis of euglycemic DKA. Given the diagnosis, her SGLT-2 inhibitor therapy was discontinued in accordance with DKA management protocols.

**Table 1:** Significant laboratory findings from venous blood gas (VBG), comprehensive metabolic panel (CMP), latent autoimmune diabetes in adults (LADA) autoimmune studies, and other laboratory tests obtained in the emergency department.

Labs	Value	Reference Range
VBG		
pH	7.20	7.32-7.42
Lactate (mmol/L)	1.9	0.9-1.7
HCO3 (mmol/L)	9	22-29
CMP		
Na (mmol/L)	131	136-145
K (mmol/L)	3.9	3.4-5.1
Cl (mmol/L)	97	98-107
CO2 (mmol/L)	9	22-29
AGAP (mmol/L)	25	6-14
Miscellaneous Labs		
B-Hydroxybutyrate (mg/dL)	63	0.2-2.8
Serum Ketones	Moderate	None
Procal (ng/mL)	1.37	≤0.10
LADA Autoimmune Studies		
C-Peptide	1.9	1.1-4.4
IA-2	<7.5	≤7.5
ZnT8	30	≤15
GAD-95	<5	≤5

At the time of admission, the patient reported full compliance with her prescribed medications, including Synjardy (empagliflozin/metformin). Notably, she was not on insulin therapy at home. Her most recent hemoglobin A1c (HbA1c) was 8.5%.

Between 2023 and 2025, she attended eight diabetes follow-ups, with documentation confirming medication adherence. She had previously trialed a glucagon-like peptide-1 (GLP-1) receptor agonist but discontinued it because of intolerable gastrointestinal side effects, including nausea and vomiting. As part of her diabetes management, she was referred to both nutrition services and a certified diabetes educator. She was repeatedly counseled on diet and exercise, including a low-carbohydrate, low-fat, low-sodium diet and engaging in at least 150 minutes of weekly physical activity. Despite these interventions, her HbA1c remained between 8.5 and 9.3% over two years, as shown in Table 2. 

**Table 2:** Hemoglobin A1c prior to hospitalization.

Lab Value	Date	Value	Reference Range
Hemoglobin A1c	02/28/25	9.0%	4.5-5.6%
	01/28/25	8.5%	4.5-5.6%
	05/13/24	9.8%	4.5-5.6%
	09/06/23	8.6%	4.5-5.6%
	03/31/23	9.3%	4.5-5.6%

Given ongoing hyperglycemia despite appropriate therapy, LADA was considered. During the hospital course, autoimmune testing demonstrated a normal C-peptide level of 1.9, negative glutamic acid decarboxylase-65 (GAD-65) antibodies (<5), and negative islet antigen-2 (IA-2) antibodies (<7.5), but a positive zinc transporter 8 (ZnT8) antibody level of 30, supporting the diagnosis of LADA as summarized in Table 1. In accordance with hospital protocol, a referral to Endocrinology was made for further evaluation and long-term management. The patient was discharged on basal insulin therapy with insulin glargine 10 units nightly. At two follow-up visits after discharge, she reported improved glycemic control, with a fasting glucose averaging approximately 150 mg/dL, improved from prehospitalization levels.

## Discussion

In 2019, the World Health Organization reclassified LADA as “slowly evolving, immune-mediated diabetes of adults,” with the term latent originally introduced to distinguish these slowly progressive cases from classic type 1 diabetes mellitus (T1DM) [4]. LADA closely resembles adult-onset T1DM but typically presents more commonly with features of metabolic syndrome, a single GAD autoantibody, and relatively preserved β-cell function at diagnosis [5]. It most commonly occurs in adults initially diagnosed with T2DM who subsequently test positive for pancreatic autoantibodies targeting islet cell cytoplasmic antigens, including GAD, IA-2, or ZnT8. Individuals with LADA generally do not require insulin therapy at the time of diagnosis and are initially managed with lifestyle modification and oral hypoglycemic agents; however, they typically progress to insulin dependence more rapidly than patients with conventional T2DM [6].

A similar autoimmune subtype has also been identified in children and adolescents who present clinically with T2DM but have pancreatic autoantibodies. This form is referred to as Latent Autoimmune Diabetes in Youth [7]. Timely and accurate diagnosis of LADA is important for guiding treatment and follow-up. 

The Immunology of Diabetes Society has proposed standardized diagnostic criteria, which include the following [8]: 1) age at onset is typically ≥30 years, 2) presence of at least one positive diabetes-related autoantibody, and 3) no requirement for insulin treatment within the first six months after diagnosis.

When testing for autoantibodies, GAD antibodies are usually measured first. If the GAD test is negative, additional testing should be performed for IA-2 and/or ZnT8 antibodies, if available. Insulin autoantibodies (IAA) may also be tested, but are only informative in patients who have not yet received insulin therapy [9].

Treatment should be tailored based on the degree of residual pancreatic beta-cell function, typically assessed using serum C-peptide levels. If the C-peptide level is > 0.7 nmol/L, indicating preserved insulin secretion, the patient may be managed according to the T2DM guidelines. However, periodic monitoring of C-peptide levels should be obtained to assess for progressive beta-cell failure.

Conversely, a C-peptide level < 0.3 nmol/L, suggesting insulin deficiency, should warrant a comprehensive insulin regimen. For values between 0.3 and 0.7 nmol/L, treatment mirrors a T2DM regimen, using agents such as metformin, GLP-1 receptor agonist, and SGLT-2 inhibitors; however, insulin should be considered early if HbA1c remains above goal. Sulfonylureas should be approached with caution in this population due to a theoretical risk of accelerated beta-cell decline [10].

SGLT-2 inhibitors offer cardiovascular and renoprotective benefits; however, their use should be carefully considered in this population. Patients with LADA who are treated with SGLT2 inhibitors may have an increased risk of DKA, as illustrated in this case. Although the exact mechanism is not fully understood, SGLT2 inhibitors are thought to contribute to ketoacidosis by increasing glucagon levels and potentially promoting hepatic ketogenesis. Additionally, they may reduce ketone clearance and increase fatty acid oxidation.

Owing to these effects, individuals with underlying insulin deficiency, such as those with LADA, are at a greater risk for developing euglycemic DKA than patients with typical T2DM [3].

As LADA progresses, patients experience declining insulin production, which increases their risk of DKA, similar to the elevated risk observed in individuals with T1DM, who rely on exogenous insulin. However, more research is needed to directly compare the incidence of DKA between LADA and T1DM patients. Many patients with LADA are initially misdiagnosed and inadequately treated as having T2DM, leading to persistently elevated blood glucose and HbA1c levels, which may further heighten the risk of DKA.

Overall, our case aligns with existing literature in that patients with LADA have a predisposition to euglycemic DKA when treated with SGLT-2 inhibitors. Additionally, it expands upon prior reports by illustrating that DKA in LADA can occur even in the presence of modest hyperglycemia and preserved renal function, emphasizing the need for heightened clinical suspicion in similar presentations.

## Conclusions

This case emphasizes the importance of recognizing LADA in adults with unexplained DKA or inadequate response to standard T2DM therapy. Given the potential for SGLT2 inhibitors to precipitate DKA in insulin-deficient states, early autoantibody testing, C-peptide evaluation, and timely insulin initiation are essential to optimize outcomes.
